# Hemolytic disease of the fetus and newborn—a perspective of immunohematology

**DOI:** 10.1016/j.htct.2024.04.122

**Published:** 2024-08-18

**Authors:** Mirelen Moura de Oliveira Rodrigues, Denise Mattos, Silvana Almeida, Marilu Fiegenbaum

**Affiliations:** a Departamento de Ciências Básicas da Saúde, Universidade Federal de Ciências da Saúde de Porto Alegre (UFCSPA), Porto Alegre, RS, Brazil; b Grupo Hospitalar Conceição (GHC), Serviço de Hemoterapia, Porto Alegre, RS, Brazil

**Keywords:** Hemolytic disease of the fetus and newborn, Alloimmunization, Immunohematology

## Abstract

**Background:**

Hemolytic disease of the fetus and newborn is a public health problem caused by maternal-fetal incompatibility; no prophylaxis is available for most alloantibodies that induce this disease. This study reviews the literature regarding which antibodies are the most common in maternal plasma and which were involved in hemolytic disease of the fetus and newborn.

**Method:**

Seventy-five studies were included in this review using a systematic search. Two independent authors identified studies of interest from the PubMed and SciELO databases.

**Main results:**

Forty-four case reports were identified, of which 11 babies evolved to death. From 17 prevalence studies, the alloimmunization rate was 0.17 % with 161 babies receiving intrauterine transfusions and 23 receiving transfusions after birth. From 28 studies with alloimmunized pregnant women (7616 women), 455 babies received intrauterine transfusions and 21 received transfusions after birth.

**Conclusion:**

Rh, Kell, and MNS were the commonest blood systems involved. The geographical distribution of studies shows that as these figures vary between continents, more studies should be performed in different countries. Investing in early diagnosis is important to manage the risks and complications of hemolytic disease of the fetus and newborn.

## Introduction

Hemolytic disease of the fetus and newborn (HDFN) is caused by maternal-fetal incompatibility when the mother has an antibody (IgG subclass) against an antigen expressed on the fetal red blood cell (RBC) and this antibody crosses the placenta.^1^ Alloantibodies usually form after exposure to non-self-antigens due to a transfusion or transplantation, or when a pregnancy results in sensitization creating antibodies against RBC antigens.^2^ HDFN has different degrees of complications that can be classified as mild, moderate, or severe. The physiopathology results in various complications such as anemia, aplastic anemia, hyperbilirubinemia, fetal hydrops, kernicterus, and death.^3^

Different maternal alloantibodies can cause HDFN, the most common are anti-D and other Rh antibodies, and Kell.^1^ However, antibodies against high prevalence antigens can also cause severe HDFN, as in the case report described by Levitt et al.^4^ The administration of prophylactic anti-D immunoglobulin in RhD negative women after delivery of a RhD positive child significantly reduced the incidence of HDFN related to anti-D in high-income countries after the 1960s.^1^^,^^5^ However, in less developed countries, HDFN still is a significant problem.^6^ Pregnant women classified as RhD positive for the D variant (partial D phenotype) who do not receive immunoglobulin prophylaxis may develop anti-D antibodies.^7^

The International Society of Blood Transfusion (ISBT) recognizes 43 different blood group systems and 378 RBC antigens of which 345 are in the blood group system.^8^ No prophylaxis is available for antibodies from other blood groups.^1^ Thus, identifying the antibodies that cause HDFN and the most common alloantibodies in populations could direct new research and the development of alloantibody prophylaxis. In addition, it is important to monitor fetuses and babies with any chance of developing HDFN and correctly identify the alloantibodies involved in HDFN. This study reviews the literature regarding which antibodies are the most common in maternal plasma and which are involved in hemolytic disease of the fetus and newborn.

## Material and methods

### Eligibility criteria and literature search

A systematic search performed by two independent authors extracted studies from the MEDLINE (accessed by PubMed) and Scientific Electronic Library Online (SciELO) databases. The terms used in the search were ‘hemolytic disease of the fetus and newborn’, ‘alloimmunization’, ‘isoimmunization’, ‘hydrops fetalis’, ‘fetal hemolytic anemia’ using the functions ‘AND’ and ‘OR’. Additionally, the exact words and synonyms were used as text words for searches in titles and abstracts. The search was restricted to studies published between 2013 and 2023. A total of 1690 results were found.

After reading the title and abstract, 1589 studies were excluded. After reading these papers, 75 were selected for data collection. To be considered eligible, case reports should present at least one case of HDFN (presence of alloantibodies causing anemia) identified by the study authors. For other study types (cross-sectional, case-control, cohort), the presence of maternal alloimmunization was accepted even if the study did not mention the clinical outcome of the babies or the development of HDFN but only mentioned the risk of HDFN. Case reports without HDFN were excluded even if alloimmunization was present. Case-control, cross-sectional and cohort studies were excluded when alloantibodies were not identified and when the study was only about phenotyping and/or genotyping pregnant women without alloimmunization.

## Results

Table 1 shows the data collected from case-report studies,^4^^,^^7^^,^^9^, ^10^, ^11^, ^12^, ^13^, ^14^, ^15^, ^16^, ^17^, ^18^, ^19^, ^20^, ^21^, ^22^, ^23^, ^24^, ^25^, ^26^, ^27^, ^28^, ^29^, ^30^, ^31^, ^32^, ^33^, ^34^, ^35^, ^36^, ^37^ Table 2 presents data from prevalence studies,^3^^,^^5^^,^^6^^,^^38^, ^39^, ^40^, ^41^, ^42^, ^43^, ^44^, ^45^, ^46^, ^47^, ^48^, ^49^, ^50^, ^51^ and Table 3 presents data from alloimmunized pregnant women.^1^^,^^11^^,^^52^, ^53^, ^54^, ^55^, ^56^, ^57^, ^58^, ^59^, ^60^, ^61^, ^62^, ^63^, ^64^, ^65^, ^66^, ^67^, ^68^, ^69^, ^70^, ^71^, ^72^, ^73^, ^74^, ^75^, ^76^, ^77^ A total of 75 studies were reviewed, 31 case reports, 19 cohorts, 22 cross-sectional, and three case-control studies. One study was classified both as a case report and a review and was included in both Table 1, Table 3.

**Table 1:** RBC alloantibodies in case-report studies.

Case	First author, year	Country	Pregnant woman				Infant								
			P	Previous histories of antibody screening	Antibody	Titer	DAT	Highest Bil	Lowest Hb	Major complaint	Intervention				outcome
								(mg/dL)	(g/dL)		RBC			Other	
											Intrau Tr	Ex tr	Tr		
1	Usman et al.^9^	Malaysia		Na	anti-E	na	+	23.45	6.2	jaundice, severe anemia, thrombocytopenia		1*		IVIG, photo	alive
2				Na	anti-E	na	+	na	10.2	jaundice, mild anemia			1	IVIG, photo, 1 fresh frozen plasma transfusion	alive
3	Pitan et al.^10^	Ireland		Yes	anti-S	64	+	9.59	5.23	hepatosplenomegaly, hypocalcemia		1		IVIG, photo, calcium infusion, and platelet transfusion	alive
4	Unterscheider et al.^27^	Ireland	P3	yes	anti-K	512	na	na	Na	severe anemia	1				death
5	Yasuda et al., 2014^11^	Japan		Yes	anti-M	4	–	17.00	6.7	severe anemia			6	IVIG, photo, corticosteroid	alive
6	Reddy and Kohan^12^	Australia		Yes	anti-S	na	+	11.70	8.1			1		IVIG, photo, platelet transfusion	alive
7	Arora et al.^13^	India	T1	Na	anti-M	32	–	na	Na	jaundice, prolonged anemia			3	photo	alive
8			T2	Na		32	–	na	Na	jaundice, prolonged anemia			2	photo	alive
9	Houston et al.^28^	Canada	P4	No	anti-D, -C	1096	na	na	Na	hydrops fetalis	2				death
10			P5	Yes	anti-D, -C	2048 (anti-D), 4 (anti-C)	na	180	18	hydrops fetalis, severe anemia	6			photo	alive
11	Kamei et al.^29^	Japan	P5	Yes	anti-D	512	+	Na	11.2			1		photo	alive
12	Zineb et al.^30^	Maroc		Yes	anti-D	na	na	na	6	hepatomegaly, hydrops fetalis		1			death
13	Mittal et al.^14^	India		Na	anti-Jk^a^	64	+	20.50	Na					photo	alive
14	Mattaloni et al., 2017^15^	Brazil		Yes	anti-Ku	na	na	Na	na	jaundice, mild anemia				photo	alive
15	DeMoss et al.^16^	USA		Yes	anti-K, anti-C, anti-e	1024 (anti-K)	+ (anti-K and anti-C)	13.19	6		3		1	photo	alive
16	Li and Blaustein^17^	USA		Yes	anti-D and anti-G	na	+	4.70	4.8	jaundice	2	3*	3	IVIG, photo	alive
17	Yousuf et al.^88^	Malaysia		Yes	anti-D, anti-G, anti-C	512	na	13.59	9.7	mild anemia				photo	alive
18	Venkataraman and Yusuf^19^	Canada	P8	Yes	anti-SARA	na	na	na	na	severe anemia		1			alive
19			P10		anti-SARA	na	+	17.00	na	jaundice		2		IVIG, photo, ventilatory support	alive
20	Quantock et al.^7^	Australia	P1	Na	anti-D, anti-E, anti-A	na	+	16.90	na	jaundice				photo	alive
21			P2	Na	anti-D	256	+	15.14	na	mild anemia				IVIG, photo	alive
22	Rauch et al.^20^	Germany		No	anti-Rd	256	+	na	3	severe anemia	3		2	ventilatory support,	alive
23	Hubinont et al.^21^	Belgium	P1	Na	anti-M	256	na	na	na	hydrops fetalis					IUD
24			P2	Yes	anti-M	na	na	na	na	hydrops fetalis					IUD
25			P3	Yes	anti-M	na	na	na	na	severe fetal anemia	1				IUD
26			P4	Yes	anti-M	2048	na	na	10.9	mild anemia				photo	alive
27	Colpo et al.^31^	Italy	P2	No	anti-D	2048	na	na	na	hydrops fetalis					IUD
28			P3	Yes	anti-D	2048	na	na	na	spontaneous abortion					IUD
29			P4	Yes	anti-D	4096	+	13.68	5.7	severe fetal anemia	1	1	2	IVIG, photo	alive
30	Bullock et al.^22^	UK		Na	anti-H	4000	+	2104.00	19.0					photo	alive
31	Millard et al.^23^	Australia		Na	anti-ATML	na	+	na	4.5	cardiac failure, pleural effusion, generalized edema		2	4		alive
32	Levitt et al.^4^	USA		Yes	anti-Ge3	256	+	6.60	6.1	mild intrauterine anemia			3		alive
33	Lawicki et al.^24^	USA	case 1	Yes	anti-Jk3	128	+	7.00	11.4					IVIG	alive
34			case 2	Yes	anti-Jk3, anti-E	16	+	9.60	na					photo	alive
35	Turley et al.^25^	Canada	P2	No	anti-D	na	na	na	na						alive
36			P3	Yes	anti-D	na	na	na	na						IUD
37			P4	Yes	anti-D	32	+	11.52	13		1	1*			alive
38	Lee et al.^32^	UK	P2	No	anti-E	8	+	7.84	6.4	severe anemia, moderate hypoxic-ischemic encephalopathy, jaundice, hepatomegaly.		1		photo	alive
39	Mandal et al.^37^	India		Yes	Anti-E, anti-Jk^a^	8 (anti-E) and 2 (anti-Jka)	na	na	na	respiratory distress					alive
40	Novoselac et al.^26^	Croatia		Yes	anti-K	32	+	6.55	na	severe anemia, jaundice			2	photo	alive
41	Riis et al.^33^	Denmark	P1	No	anti-D	16,000	+	27.01	7.25	severe anemia, jaundice, hepatosplenomegaly			1	photo	alive
42	Souabni et al.^34^	Maroc	P3	Yes	anti-D	na	na	na	5.8	severe anemia					death
43	Moreno et al.^35^	Spain		No	anti-Kp^a^	16	+	na	4.2	severe anemia, hydrops fetalis			1		death
44	Fives et al.^36^	USA		No	anti-Go^a^	na	+	18.8	6.7	jaundice			2	photo	alive

DAT, direct antiglobulin test; Bil, bilirubin; Hb, hemoglobin; photo, phototherapy; Intrau Tr, intrauterine transfusion; Ex tr, exchange transfusion; Tr, transfusion; IVIG, intravenous immunoglobulin; +, positive; -, negative; IUD, intrauterine death; P, pregnancy; T, Twin; np, not performed;.

⁎ double volume exchange transfusion; na, not available.

**Table 2:** Frequency of RBC alloantibodies in prevalence studies.

Study	Study type	Country	Total pregnancies (n)	Presence of alloantibody		HDFN (n)	Intervention^b^				Outcome
				Pregnancy (n)	(%)		IUT	Ex Tr	Tr	Photo	
Altuntas et al.^38^	CS	Turkey	4840	65	1.34	30	3			30	3 hydrops fetalis, 1 death
Hassan et al. a^39^	CS	Malaysia	5163	30	0.58	14		1	1	14	1 hydrops fetalis
Velkova^40^	CS	Macedonia	22,009	205	0.93	48					2 deaths
Mbalibulha et al.^47^	CS	Uganda	726	88	12.12						
Krstic et al.^41^	CS	Croatia	102,982	184	0.18	3			1	2	
Sidhu et al.^42^	CS	India	750	15	2.00						
Girault et al.^48^	CS	France	113	78	69.00	78	78				
Peeters et al.^43^	CS	Belgium	9419	46	0.49	25	1			1	1 death
Zonneveld et al.^6^	CS	Suriname	214	19	8.87	11		2		4	
Chatziantoniou et al.^5^	CS	UK	46,182	130	0.28	65	6	9		19	8 IUD
Slootweg et al.^44^	cohort	Netherlands	3200,000	1026	0.000003	49	48	1			3 IUD
Kahar^45^	CS	India	1960	20	1.02						
Matteocci et al.^46^	CS	Italy	28,089	3000	11.00	81					
Moinuddin et al.^49^	CS	USA	4545	34	0.74						
Rahimi-Levene et al.^3^	CS	Israel	90,948	900	0.99	17	2	2	13		1 death
Özköse et al.^50^	CC	Turkey	37,344	153	0.40	49	23	8	8	45	4 IUD
Ali et al.^51^	CS	Sudan	130	14	10.77						
**total**			**3555,414**	**6007**	**0.17**	**470**	161	23	23	115	

%, rate of n pregnant with antibody;.

CS: Cross-sectional; CC: Case-control; IUT: intrauterine transfusion; Ex tr: exchange transfusion; Tr: transfusion; Photo: phototherapy; IUD: intrauterine death.

a 9 cases of intravenous immunoglobulin.

b Some cases of HDFN have not received clinical intervention or the study does not mention the intervention.

**Table 3:** Studies with alloimmunized pregnant women, HDFN, and intervention.

			Presence of alloantibody			Intervention			
Study	Study type	Country	n pregnant	(%)	n HDFN	IUT	Ex Tr	Tr	Photo
Rath et al.^59^	cohort	Netherlands	393	100	393	70		93	
Smits-Wintjens et al.^60^	cohort	Netherlands	347	100	347	240	134	255	
Tiblad et al.^61^	cohort	Sweden	290	100	108	37	46		94
Doyle et al.^52^	cross sectional	Ireland	1106	100	62	62			
Kapur et al.^53^	cross sectional	Netherlands	70	100					
Yasuda et al.^11^	review	Japan	34	100	34				
Verduin et al.^54^	cohort	Netherlands	260	100	287	287			
Garabedian et al.^63^	cohort	France	81	100	81	81	28	42	70
Garabedian et al.^62^	cohort	France	77	100	77	77			
Philip et al.^89^	cross sectional	India	42	100	42	42		13	
Kristinsdóttir et al.^55^	cross sectional	Iceland	375	100	179		8		179
Stetson et al.^56^	cross sectional	USA	146	100	0				4
Sonneveld et al.^57^	cohort	Netherlands	679	100	37			13	24
Zwiers et al.^1^	case-control	Netherlands	1326	100	232				
Phung et al.^58^	cross sectional	France	106	100	106	106			
Healsmith et al.^65^	cohort	Australia	115	100	59	11	6		59
Sánchez-Durán et al.^66^	cohort	Spain	337	100	103	45	24	21	38
Snelgrove et al.^67^	cohort	Canada	232	100	232	232			131
Crawford et al.^68^	cohort	UK	11	100	11	11			
Gudlaugsson et al.^69^	cohort	Iceland	130	100	35	5	13	7	32
Lieberman et al.^70^	cohort	Canada	128	100	21	2	2	7	17
Şavkli et al.^71^	cohort	Turkey	42	100	42	42			
Liu et al.^72^	cohort	Sweden	1079	100	157	87	52		216
Maisonneuve et al.^73^	case-control	France	41	100	41	41	4		
Lee et al.^74^	cohort	USA	36	100	35	36			
Vlachodimitropoulou et al.^75^	cohort	Canada	128	100	128	128	12	26	54
Ghesquière et al.^76^	cohort	France	207	100	126	105	53	92	133
Kureba et al.^77^	cross sectional	Ethiopia	98	100	21	21	15	4	20
**Total**			**7916**		**2996**	**1768**	**397**	**573**	**1071**

Results extracted from case reports included 47 cases with antibodies, but three cases were excluded because the babies did not show any signs of HDFN, one with anti-H,^22^ and two with anti-Jk3.^24^ Of the 44 cases with HDFN, 26 women had already been screened for antibodies prior to the reported pregnancy. The antibodies implicated as causing HDFN were anti-D (10 cases in isolation and 5 with associated antibodies), anti-E (3 cases in isolation and 3 cases with associated antibodies), anti-M (6 cases), anti-Jk3 (1 in isolation and 1 with associated antibodies), anti-Jk^a^ (1 in isolation and 1 with associated antibodies), anti-G (2 cases with associated antibodies), anti-K (2 in isolation and 1 with associated antibodies), anti-S (2 cases), anti-SARA (2 cases), anti-C (4 cases with associated antibodies), anti-e and anti-A (1 case with associated antibodies each) and anti-Ku, anti-Go^a^, anti-Ge3, anti-Kp^a^, anti-Rd, anti-ATML and anti-H with each one in isolation. The most observed complications reported were severe anemia (13 cases), mild anemia (6 cases), and jaundice (12 cases). Of the 44 cases, 11 evolved to death. Of the anti-D cases, two women were D partial^7^^,^^25^ responsible for 5 cases of anti-D HDFN. A total of 20 IUTs were performed in 9 fetuses, 16 exchange transfusions in 12 babies and 33 transfusions in 14 babies. Phototherapy were necessary as treatment for 25 patients.

Table 2 presents 17 prevalence studies (15 cross-sectional, 1 cohort, and 1 case-control). A total of 3555,414 pregnant women were evaluated; of these 6007 women presented alloantibodies in the gestational period, corresponding to a 0.17 % alloimmunization rate. HDFN was present in 470 babies, of these 161 babies received at least one IUT, 23 received transfusions, 23 performed exchange transfusions, and 115 received phototherapy. Four babies had hydrops fetalis, and 20 evolved to death (15 intrauterine deaths).

For alloimmunized pregnant women, 28 studies were included (1 review, 2 case-control, 7 cross-sectional, and 18 cohort). A total of 7911 women presented alloantibodies in the gestational period. Almost one third (2996) of the babies had HDFN and of these 1768 babies received at least one IUT, 397 received exchange transfusions, 573 received transfusions, and 1071 received phototherapy.

Details of the single or multiple maternal antibodies identified are reported in Table 4; multiple antibodies were found in 1791 cases. Nineteen different alloantibodies which cause HDFN were cited in case reports. ABO incompatibility was found in 3307 cases.

**Table 4:** RBC antibodies present in maternal plasma.

Antibody	Case report (Table 1)	Prevalence studies (Table 2)	Alloimmunized pregnant women (Table 3)	Antibody	Case report (Table 1)	Prevalence studies (Table 2)	Alloimmunized pregnant women (Table 3)
	n	n	n		n	n	n
Anti-D	11	821	3366	anti-C, -G; -K		1	
Anti-D, -C	2	24	354	anti-c		85	543
anti-D, -c			3	anti-c; -K			5
anti-D, -E		7	45	anti-c; -K; -Jk^a^; -M			1
anti-D, -C, -E		1	31	anti-c; -Le^a^			1
anti-D, -E; -K		1	1	anti-cE			12
anti-D, -Jk^a^			7	anti-cE; -Jk^a^			1
anti-D; -S		2		anti-ce			1
anti-D; -Le^a^		1	3	anti-Ce			1
b anti-D and multiple antibodies		36	104	anti-c, -C			1
anti-D, -C, -G	1	1	2	anti-c, -C^w^			1
anti-D, -G	1		2	anti-c; -Jk^b^			1
anti-D, -E; -A	1			anti-c, -E; -Fy^a^			1
anti-D, -Ce		13		anti-c, - E; -Jk^a^		1	1
anti-D, -cE		1		anti-c, -E; -K		2	
anti-D, -Cc		1		anti-c; -Fy^a^; -K		1	1
anti-D; -K		1		anti-c, -E; -Lu^a^		1	
anti-D; -Cce		5		anti-c; -s		1	
anti-D, -C; -Jk^a^			8	anti-c; -Fy^a^		2	2
anti-D; -K			4	anti-c, -E; -Le^b^; -M		1	
anti-D, -C; -M			2	anti-c; -S		1	
anti-D, -C; -Kp^a^			2	anti-c; -Jk^a^		2	2
anti-D; -Fy^a^			1	anti-E	3	182	1130
anti-D, -C; -Jk^b^			1	anti-E; -K		6	3
anti-D, -C; -K			3	anti-c, -E		49	46
anti-D, -C; -Fy^b^			1	anti-E, -C^w^		4	1
anti-D, -C; -Kp^a^			1	anti-E; -Fy^a^		2	3
anti-D, -C, -Fy^a^			5	anti-E; -Jk^a^	1	4	4
anti-D, -E; -Fy^a^			1	anti-E; -Le^a^		2	
anti-D, -E; -Jk^a^			1	anti-E; -Le^b^		1	
anti-D; -Jk^a^; -S			1	anti-E; -Lu^a^		1	1
anti-CD			9	anti-E; -P1		1	
anti-CD; -K			1	anti-E; -M		2	
anti-D, -E, -G			1	anti-E; -K; -C^w^		1	
anti-D; -s			2	anti-E; -Fy^a^; -K		1	
anti-D, -C; -S			4	anti-E; -S; -Le^a^		1	
anti-D; -Fy^a^; -Jk^a^			1	anti-E; -Fy^a^, -Fy^b^; -K; -S	1		
anti-D, -C, -E; -Fy^a^			1	anti-E, -C^w^; -Fy^a^; -S; -Jk^a^	1		
anti-D; -K; -M			1	anti-E, -f; -K; -Le^a^; -Jk^b^		1	
anti-C		20	27	anti-E; -S			1
anti-C; - K		1		anti-E; -s			1
anti-C, -C^w^		2	156	anti-e		6	29
anti-C, -G		1		anti-e, -Ce			1
anti-C, -e		6	7	anti-e; -S			1
anti-C, -E		2		b anti-E or anti-c			10
anti-C; -Jk^b^		2		anti-C^w^		37	3
anti-C; -Le^a^		1	1	anti-C^w^; -P1		1	
anti-C; -Lu^a^		1		anti-C^w^, -Le^a^		1	
anti-C; -Jk^a^			2	anti-C^w^; -Fy^a^			1
anti-C; -Fy^a^			1	anti-C^w^; -S			1
anti-C, -E; -K		2		anti-f		1	2
anti-C, -K, -e	1	1		anti-Go^a^	1		
anti-C, - E; -Le^a^		1		a anti-Le^a^		108	
anti-C; -K; -Le^a^		1		a anti-Le^a^; -M		1	
anti-K	2	1176	805	a anti-Le^b^		34	
anti-K; -U		1		a anti-Le^a^, -Le^b^		14	26
anti-K; -Le^a^		2	1	a anti-Le^a^, -Le^b^; -Lu^a^		1	
anti-K; -Le^b^			1	anti-S	2	16	19
anti-K; -Le^a^, -Le^b^		1		anti-S, -U		1	
anti-K; -Jk^a^		2	1	anti-s		2	1
anti-K -Jk^b^		1		anti-U		1	5
anti-K; -Fy^a^		3		anti-M	6	104	207
anti-K; -Jk^a^; -S		1		anti-M; -Lu^a^		1	
anti-K, -Kp^a^		2		anti-N		6	1
anti-K; -C^w^		2		anti-SARA	2		
anti-K; -Bg^a^		1		b anti-Jk			93
anti-K; -Kn^a^		1		anti-Jk^a^	1	22	19
anti-K or -Lu^b^		5		anti-Jk^a^; -Cr^a^		1	
anti-k		3		anti-Jk^a^; -Le^a^		1	
anti-Ku	1			anti-Jk^b^		9	5
anti-k; -Lu^b^		1		anti-Jk3	1		
anti-Kp^a^	1	16	1	anti-Jk3; -E	1		
anti-Kp^a^; -Le^b^		1		anti-Lu^a^		35	2
anti-Kp^a^; -P1		1		anti-Lu^b^		1	2
anti-Kp^b^		1		a anti-Ch^a^		1	
b anti-Fy			129	a anti-Bg^a^		5	
anti-Fy^b^		6	3	a anti-Rd	1		
anti-Fy^b^; -M		1		anti-Cr^a^		4	
anti-Fy^a^		12	55	anti-ATML	1		
anti-Fy^a^; -M			1	anti-Ge3	1		
anti-Fy3		1		anti-H	1	1	
anti-P1		14	1	anti-Chido		1	1
anti-Yt^a^		6		anti-Jr^a^		1	1
anti-Wr^a^		1	1	anti-Vel		1	2
ABO incompatibility		3000	291	ABO incompatibility and other antibodies		15	
Antibody not indicated or unidentified^b^		5	468				
**Total**	**43**	**5999**	**8133**				

a Rarely implicated in HDFN.

b unspecified Important: Lewis's antibodies are not implicated in HDFN.

**Figure 1:**
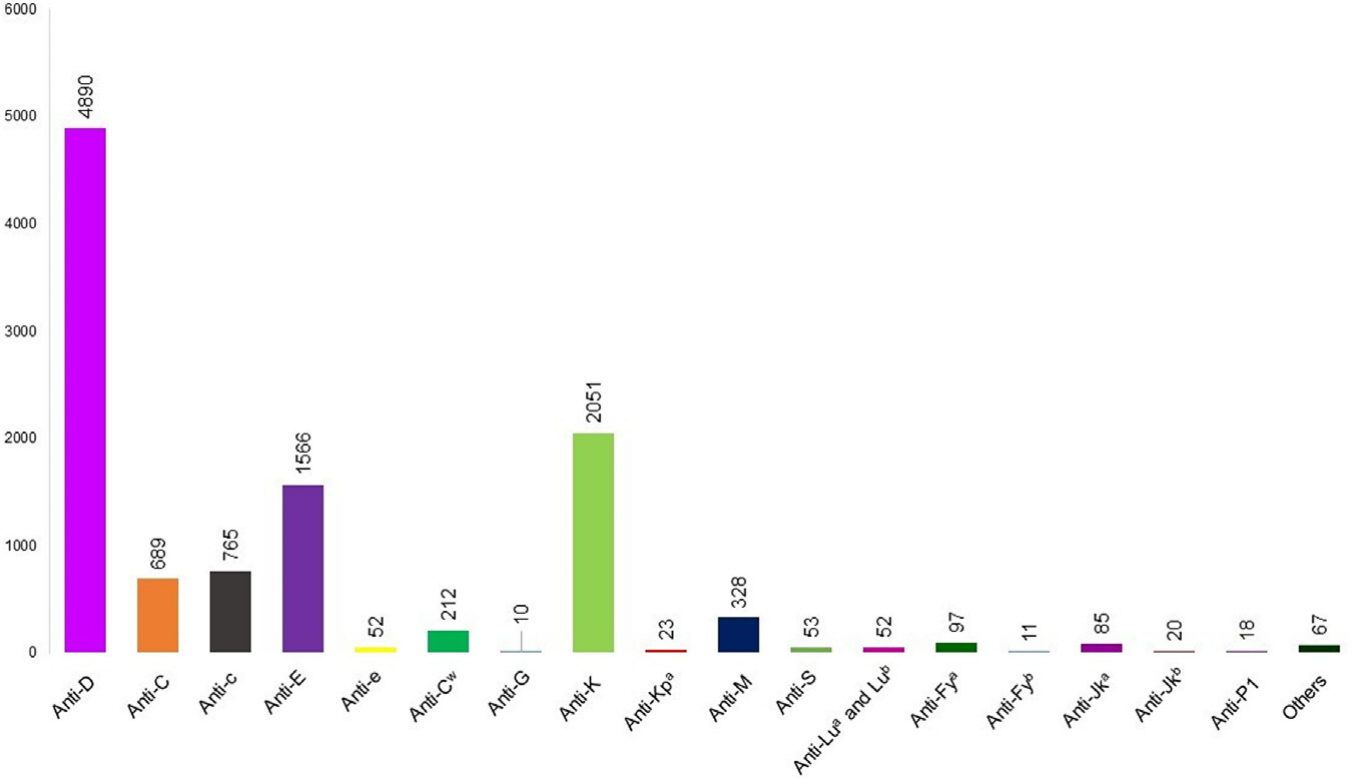
RBC antibodies present in maternal plasma. anti-N (*n* = 7), anti-U (*n* = 7), anti-Yt^a^ (*n* = 6), anti-Bg^a^ (*n* = 6), anti-s (*n* = 5), anti-Cr^a^ (*n* = 5), anti-f (*n* = 4), anti-k (*n* = 4), anti-Vel (*n* = 3), anti-Jk3 (*n* = 2), anti-SARA (*n* = 2), anti-H (*n* = 2), anti-Chido (*n* = 2), anti-Jr^a^ (*n* = 2), anti-Wr^a^ (*n* = 2), anti-Go^a^, anti-Kn^a^, anti-Ku, anti-Kp^b^, anti-Ch^a^, anti-Rd, anti-ATML, anti-Ge3 (*n* = 1 each).

**Figure 2:**
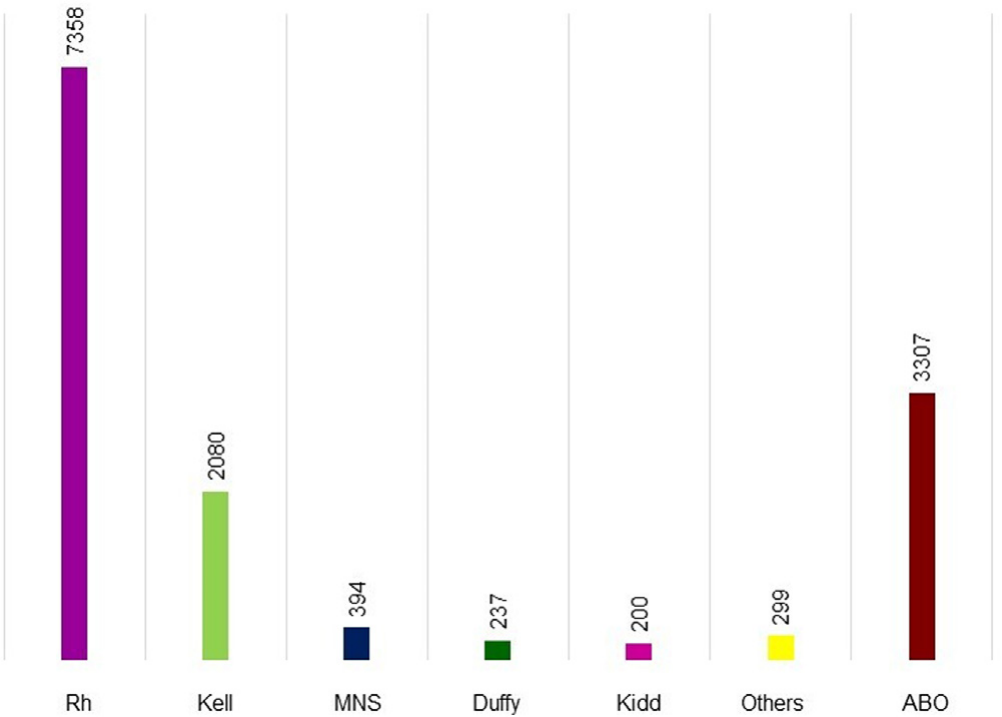
RBC blood group system present in maternal plasma.

Figure 1 shows the alloantibodies found in isolation or combined with other antibodies. Anti-D (4890 cases), anti-K (2051 cases), and anti-E (1566 cases) were the most common in maternal plasma. Figure 2 shows the antibodies against RBC antigens classified according to the blood group system. Rh was the commonest with 7358 cases, followed by Kell (2080 cases), MNS (394 cases), Duffy (237 cases), and Kidd antibodies (200 cases). Anti-Lewis is not mentioned in Figures 1 or 2 because anti-Lewis antibodies are not implicated in HDFN.^78^

## Discussion

HDFN is an important public health problem due to maternal-fetal incompatibility, which occurs due to fetal or neonatal hemolysis caused by IgG antibodies that cross the placenta.^2^ Just the presence of alloantibodies in maternal plasma is not sufficient to cause HDFN, it is necessary that antibodies cross the placenta and the fetus or neonate have the antigen expressed on their RBCs.

This review found 13,966 reports of alloimmunized pregnant women with anti-D being the most common alloantibody (35.01 %) followed by anti-K (14.69 %), anti-E (11.21 %), anti-c (5.48 %), anti-C (4.93 %) and anti-M (2.35 %). From prevalence studies, 0.17 % of pregnant women were alloimmunized, and from studies on alloimmunized pregnant women, 36.84 % had a baby with HDFN; these figures might be higher because some studies do not mention the clinical outcome of newborns. When verifying the geographical distribution of alloantibody studies, most were performed in Europe (35 studies), America (16 studies), and Asia (15 studies). These data show the need to perform more studies in other locations, thereby contributing to the understanding of alloimmunization and HDFN, for example, which antibody is the most involved in HDFN.

The Rh blood system is a highly polymorphic blood group^79^ with 56 antigens having been described.^80^ The most important antigens are D, E, e, C, c. Rh antibodies were found in the plasma of 7358 (52.69 %) of the 13,966 pregnant women, either in isolation or with other antibodies. Anti-E was the second most common alloantibody of the Rh system, followed by anti-c, anti-C, anti-C^w^, anti-e, anti-G, anti-f and anti-Go^a^. The anti-G alloantibody is important for the correct identification of this latter alloantibody and differentiation from the anti-C and anti-D alloantibodies because if a pregnant woman does not present anti-D, prophylaxis is strongly recommended to avoid RhD alloimmunization.^18^^,^^28^

ABO maternal-fetal incompatibility is common because ABO antibodies develop naturally. Matteocci et al.^46^ analyzed HDFN related to ABO incompatibility: 81 babies had a positive direct antiglobulin test (DAT) and 32 required invasive treatments (exchange transfusions or intravenous immunoglobulins).^46^ When ABO incompatibility was present, the O blood group was associated with reduced alloimmunization compared to other blood group antigens because of the presence of anti-A/anti-B antibodies in maternal plasma. This could occur because of the clearance of A or B fetal RBCs in maternal plasma mediated by ABO antibodies. This clearance might avoid other alloimmunizations. Doyle et al. analyzed anti-D levels and IUT; on comparing ABO blood groups in respect to IUT, women of the A blood group have a higher risk of carrying a fetus with significant HDFN compared to women of the O blood group.^52^

Kell alloantibodies are the second most commonly related to HDFN.^44^ Anti-K is associated with a risk of HDFN, nonetheless the titer does not necessarily correlate with the clinical severity of HDFN.^26^ In a retrospective cohort that evaluated 1026 Kell immunized pregnancies, a cut-off value for risk of severe HDFN was established with 93 K-positive fetuses; the value of 4 identified a risk of severe HDFN.^44^ However, severe cases of HDFN occur with lower anti-K titers, demonstrating that there is no correlation between clinical outcome and the titer.^81^ Accordingly, any case of an anti-K positive pregnancy should be accompanied when it is not possible to predict the fetus phenotype because anti-K antibodies cause erythropoiesis suppression and the destruction of erythroid progenitors.

Anti-M is one case of alloantibodies in which IgM and IgG antibodies are found. IgG antibodies can cross the placenta and cause HDFN to variable degrees. A review of the Japanese population presented 33 cases of HDFN caused by anti-M; of those 29 developed severe HDFN, five presented IgG subclasses (IgG_1_ or IgG_3_).^11^ Six case reports related to anti-M antibodies describe babies with HDFN, three with different titers and clinical outcomes that required transfusions (RBCs and platelets), one required intrauterine transfusion for severe fetal anemia, and two evolved to intrauterine death. These data show that the severity was independent of the anti-M titer and the importance of the differentiation of IgG and IgM antibody classes.^11^^,^^13^^,^^21^

The physiopathology of anti-M is similar to those of anti-K and anti-Gerbich type 3 (anti-Ge3) with mechanisms of apoptotic or erythropoietic suppression (extracellular hemolysis) differing from the physiopathology of anti-D.^82^

Kidd blood group antigens are implicated in hemolytic transfusion reactions and HDFN. The most common antibodies are anti-Jk^a^ and anti-Jk^b^, which cause mild to severe disease. There is another antibody, anti-Jk3, which can be induced by alloimmunization in Kidd null phenotype people.^24^ Anti-Jk3 reacts with both Jk^a^ and Jk^b^ antigens.^24^ It remains unclear which anti-Jk3 titers produce a clinically significant risk of HDFN; according to Lawicki et al.,^24^ fetuses with titers of 16 or higher should be monitored.

Multiple maternal alloantibodies could represent an increased risk of developing HDFN. One study from Israel showed that 6.8 % of pregnancies with multiple alloantibodies develop severe HDFN.^3^ Phung et al. compared the mean estimated daily decrease in Hb between the first and second IUT when the pregnant woman had anti-D in isolation and when it was associated with two other antibodies. This study showed that the drop was lower when the pregnant had only anti-D.^58^ These datasets suggest that the presence of multiple maternal alloantibodies could point toward an increase in the risk of the severity of HDFN. In this review, Table 4 presents observation studies and six case reports in which the HDFN was caused by multiple alloantibodies.^6^^,^^7^^,^^16^, ^17^, ^18^^,^^24^^,^^37^^,^^38^^,^^40^^,^^43^^,^^58^

One key factor in the risk of HDFN is the antibody class; IgM antibodies are not implicated in HDFN because they are not capable of crossing the placenta however IgG antibodies cross the placenta and can cause HDFN. IgG antibodies are subclassified into IgG_1_, IgG_2_, IgG_3_, and IgG_4_. The severity of HDFN may be related to the IgG subclasses; IgG_1_ and IgG_3_ cause severe HDFN and so these parameters should be included in protocols for measuring the intensity of the HDFN.^40^

When all antigen sites are occupied by the respective antibody, the reaction of these RBCs with commercial antisera produce a false negative phenotype in the antihuman globulin phase.^26^ These cases are rare but can occur when a direct antiglobulin test is positive. Using methods without the antihuman globulin phase with saline monoclonal IgM antiserum is an option to solve these cases, but these reagents are not always available.^83^ An IgG blocking technique can be used for RBCs that have a weak to moderately positive (2+) DAT;^84^ other methods use ethylenediaminetetraacetic acid (EDTA) elution, chloroquine diphosphate (CPD) and heat elution to remove IgG antibodies from RBCs.^26^

Molecular tests present an option to solve cases of false negative phenotypes in newborns. Novoselac et al. used a genotyping test (single specific primer-polymerase chain reaction - PCR-SSP) to confirm the K*01/K*02 genotype in a baby and solve an anti-K mediated HDFN.^26^ Lawicki et al. also used this test to confirm the JK*A/JK*B genotype in a baby and solve an anti-Jk3 mediated HDFN.^24^

Knowing the molecular basis of blood group antigens is also important to predict phenotypes and the formation of null alleles that might cause a lack of expression of the phenotype. The ISBT lists all blood group antigens and genes involved in antigen expressions. The null phenotype is caused by different mutations in the genes; some have higher frequencies in specific population groups, for example, the Fy null allele (c.1-67T>C, rs2814778) in African descendants^85^ and the Jk null allele (T871C mutation) in Polynesian descendants.^86^

Sequencing tests are useful to understand unexplained discrepancies between phenotype and genotype, discover antigen variants and predict the risk and significance of alloimmunization to cause HDFN and hemolytic transfusion reactions.^25^ In order to understand the risk of HDFN, it is recommended to perform maternal and paternal phenotyping or genotyping to predict fetal phenotype.^66^ However, paternal phenotyping is not always available in cases of suspected HDFN.^24^

The screening of irregular RBC antibodies and maternal antibody titers using the indirect antiglobulin test during prenatal care is important for the correct diagnosis and early clinical intervention as is using Doppler ultrasound to measure the peak systolic blood flow velocity in the middle cerebral artery.^65^ The correct identification of maternal alloantibodies and estimated risk of the fetus carrying the RBC antigen is important in prenatal exams.^77^ The precise identification of one or multiple alloantibodies and their clinical significance help to select packed RBC units for transfusion when IUT are needed, and after birth if the newborn needs a transfusion. In the newborn, it is important to perform DAT, and when the DAT is positive, to perform elution to identify the antibody bound to the RBC membrane.^78^

Some developed countries use non-invasive prenatal testing with cell-free fetal DNA (cffDNA) circulating in the maternal plasma of pregnant women to identify the fetus genotype and estimate the risk of HDFN.^87^ RhD incompatibility using cffDNA is an efficient prevention strategy as anti-D prophylaxis will only be provided if the fetus is RhD-positive.^61^ cffDNA is useful for other RBC antigens to determine if the fetus presents antigens for which the mother has alloantibodies and determine the risk of HDFN. This method is non-invasive but it is not available in all hemotherapy services.

IUTs were needed at least once in 1938 fetuses in this review however, this data is limited because some studies did not mention fetal outcomes. IUT is an intervention related to the severity of anemia, and early IUT (before 20 weeks) is associated with a higher risk of fetal injury.^73^ Despite the risk, IUT is an important intervention to treat fetal anemia. The need for IUT should be considered in prenatal care. However, there are great differences in the prenatal care provided in different countries and some pregnant women who need an IUT do not receive it.

Unfortunately, some countries do not screen all pregnant women for alloantibodies, due to many reasons such as the absence of universal healthcare, failure to recognize events during pregnancy that could trigger alloimmunization, and geographical difficulties in accessing a hemotherapy reference center.^77^

## Conclusion

Investing in early diagnosis is important for the management of risks and complications related to the development of HDFN. Approaches using serological and molecular tests are useful for early diagnosis. Knowing the physiopathology of alloantibodies is also helpful in understanding the evolution of HDFN. Furthermore, considering the high diversity of blood group alleles, there is a need to study the frequency of pregnant alloimmunization and HDFN in different populations.

## Authors’ contributions

MMOR and MF designed the study, performed the systematic literature search, performed data interpretation, and wrote the manuscript; MMOR, DM, SA and MF analyzed the data. All authors were involved in writing the paper and approved the final version.

## Conflicts of interest

The authors declare that they have no conflicts of interest.
